# A bi-institutional observational study comparing short-term and long-term outcome of operative and non-operative management of clinical and radiological flail chest injuries

**DOI:** 10.1186/s13049-025-01400-8

**Published:** 2025-05-15

**Authors:** Eva-Corina Caragounis, Monika Fagevik Olsén, Lena Sandström, Rauni Rossi Norrlund, Lovisa Strömmer, Hans Granhed

**Affiliations:** 1 Department of Surgery, Institute of Clinical Sciences, Sahlgrenska Academy, University of Gothenburg, Gothenburg, Sweden; 2 Department of Surgery, Region Västra Götaland, Sahlgrenska University Hospital, Gothenburg, Sweden; 3 Department of Physical Therapy, Institute of Neuroscience and Physiology, Sahlgrenska Academy, University of Gothenburg, Gothenburg, Sweden; 4 Department of Physiotherapy, Faculty of Health Sciences, University of the Witwatersrand, Johannesburg, South Africa; 5 Allied Health Professionals Function, Function Area Occupational Therapy and Physiotherapy, Karolinska University Hospital Solna, Stockholm, 141 52 Sweden; 6 Division of Physiotherapy, Department of Orthopaedics, Danderyd Hospital, Stockholm, 182 88 Sweden; 7 Department of Radiology, Institute of Clinical Sciences, Sahlgrenska Academy, University of Gothenburg and Sahlgrenska University Hospital, Gothenburg, 413 45 Sweden; 8 Division of Surgery and Oncology, Department of Clinical Science, Intervention and Technology (CLINTEC) and Department of Global PublicHealth, Karolinska Institute, Stockholm, 141 52 Sweden

**Keywords:** Rib fracture, Flail chest, Chest wall injury, SSRF, Mechanical ventilator, Pain, Lung function, Long-term follow-up

## Abstract

**Background:**

Operative management of chest wall injuries requiring ventilatory support has been shown to decrease the time spent on ventilator. The main purpose of this study was to investigate whether operative management reduces the need for mechanical ventilation and the impact of surgery on long-term outcome concerning pain, lung function and movement.

**Methods:**

This is a bi-institutional prospective observational study comparing operative (Op) and non-operative (Non-Op) management of adult trauma patients with flail chest injuries. Data on the need for and LOS in intensive care (ICU), on mechanical ventilator (MV), and in hospital, and incidence of pneumonia and tracheostomy was collected. Clinical follow-up after six weeks, six months and one year concerning lung function, CT-lung volume, physical function, pain, and quality of life (QoL) was performed.

**Results:**

There was no difference in the need for (29%) and LOS on MV and in ICU between the Op and Non-Op groups. Chest wall surgery was performed 4 days (range 2–14) post trauma and associated with a longer hospital LOS. Pneumonia was more common in the Non-Op group (37% vs. 18%, *p* = 0.003). Fifty patients in the Op group and 38 patients in the Non-Op group were enrolled in a follow-up where Non-Op group experienced more pain in the first six months and had a higher daily dose of oral morphine during the first six weeks post trauma. The best residual lung function and CT-lung volume was seen in patients managed with muscle-sparing surgery without thoracotomy. No considerable difference in pain, physical activity, physical function and QoL were seen between the groups after one year.

**Conclusions:**

Operative management of flail chest injuries did not decrease the need for mechanical ventilation or the length of stay in ICU. Operating on non-ventilated patients may increase the length of hospital stay depending on day of surgery. Surgery was associated with a decreased incidence of pneumonia, less pain and subjective symptoms the first months’ post-trauma despite operated patients being older and with more severe trauma, but after one year there were no significant differences between the groups. Operative technique may influence outcome and should be studied further.

**Trial registration:**

ClinicalTrials.gov: NCT02132416, 7 May 2014.

## Background

Major thoracic trauma is associated with unstable chest wall injuries, which can lead to respiratory insufficiency [1–3]. Surgical stabilization of rib fractures (SSRF) in several randomized controlled trials (RCTs), has been shown to reduce time spent on ventilator in patients with flail chest in need of mechanical ventilation (MV) [4–6], reduce length of stay (LOS) in intensive care unit (ICU) [4–7] and in hospital [5, 8], and decrease the incidence of tracheostomy [4, 7], pneumonia [4–6] and mortality [8]. Current practice guidelines support SSRF of flail chest injuries and selectively in multiple displaced fractures in adult trauma patients [9–11]. The indication for surgery in patients with severe traumatic brain injury (TBI) and extensive lung contusions is unclear, since these patients often require prolonged MV [3, 12]. However, a multicenter retrospective study of SSRF in patients with moderate and severe TBI showed a lower rate of pneumonia and mortality in surgically managed patients [13]. Whilst, SSRF has shown benefit to ventilated patients with flail chest injuries in the short-term it is unclear if the procedure benefits non-ventilated patients both short- and long-term.

Operative management of chest wall injuries seems to decrease the level of perceived pain in the short-term both in flail [4, 6] and non-flail [14, 15] injuries. Previous studies show conflicting results concerning lung function when comparing operative and non-operative management of chest wall injury [4, 5, 7, 15]. Two studies found no difference in lung function between the groups in the first weeks and months after trauma [7, 15]. It is unclear whether surgery influences long-term outcomes concerning lung function, physical function and Quality of Life (QoL). We have previously shown, in a prospective longitudinal study, that surgically managed patients improve concerning subjective symptoms, QoL and lung function, up to a year after surgery [16].

The objective of this study was to clarify whether operative management of flail chest injuries reduces the need for mechanical ventilation. Secondary aims include LOS in ICU, on MV and in hospital, incidence of pneumonia, tracheostomy, mortality and long-term follow-up of pain, lung function, lung volume estimated by computed tomography (CT), physical function and QoL.

## Methods

### Study design

This is a prospective observational cohort study comparing operative and non-operative management of flail chest injuries between the two largest trauma centers in Sweden during 2014–2018. The primary outcome was the need for mechanical ventilation and secondary outcomes were LOS in ICU, on MV and in hospital, incidence of pneumonia, tracheostomy, mortality and long-term follow-up of pain, lung function, CT-lung volume, physical function and QoL. Flail chest was managed according to the guidelines of each hospital, operatively at Sahlgrenska University Hospital (Op group) and non-operatively at Karolinska University Hospital Solna (Non-Op group). There were no standardized protocols in place for the management of flail chest injuries in the two hospitals, at the time of the study.

Patients were invited to participate in a one-year follow-up and gave their informed consent after written and verbal information. The inclusion criteria for the study were: adult (≥ 18 years) trauma patients with flail chest defined according to Abbreviated Injury Scale (AIS) as three or more adjacent ribs each fractured in more than one location and/or paradoxical chest wall movement [17] and/or sternal flail with chondral/costal fractures in conjunction with sternal fractures [18]. Patients with severe TBI, defined as Head AIS > 3 [17] and/or in need of neurosurgery, spinal injury and pre-existing severe neurological and/or musculoskeletal disease that influence chest wall movement and lung volume were excluded. Patients that died within 24 h of admission or who were moribund or not able to undergo SSRF were excluded.

The sample size was estimated from a power analysis based on data from a previous study from our institution showing that 47% of operatively managed patients with flail chest needed ventilator support, compared to 72% of non-operatively managed historical controls [19]. The calculations showed that 59 patients needed to be included, in each group, in order to give a power of 80% with a 95% significance level. During the study period Karolinska University Hospital Solna began treating selective patients with unstable chest wall injuries operatively, therefore, new inclusion was terminated before statistical power was reached. Retrospective data on all eligible patients concerning hospital outcome during the inclusion period was retrieved and additional analyses performed.

### Surgical procedure

Surgical fixation of rib fractures was performed at Sahlgrenska University Hospital (Op group) by using the MatrixRIB™ Fixation System (DePuy Synthes, West Chester, USA), consisting of pre-shaped angular locked plates and intra-medullary splints. The operative technique changed over time. Initially patients were operated with a non-muscle sparing approach. Thoracotomy was part of the procedure and performed in order to clear out hemothorax, identify and, if necessary, manage intra-thoracic injuries. With time the operative technique became more minimally invasive with a muscle sparing approach and thoracotomy was performed selectively [16, 19]. None of the patients underwent thoracoscopy. Three surgeons performed all the operations. Broad-spectrum antibiotic prophylaxis was administered pre-operatively.

### Data collection

#### Hospital data

Patient and trauma demographics; age, sex, comorbidity, mechanism of injury (MOI), AIS for Head and Thorax [17], Injury Severity Score (ISS) [20] and New Injury Severity Score (NISS) [21] were collected and analyzed. Data on the need for and length of stay (LOS) on mechanical ventilator, in ICU and hospital was collected. Patients not being able to be extubated on the day of surgery were assessed as needing MV. Patients were offered, regardless of study group, conventional analgesia with paracetamol, non-steroidal anti-inflammatory drugs (NSAID), opioids and thoracic epidural analgesia (TEDA). Some patients in the Op group received an inter-pleural catheter peri-operatively for administration of local anaesthetic. Complications such as pneumonia, tracheostomy, re-operation, and mortality were studied. Pneumonia was diagnosed by clinical signs of chest infection, changes on radiological examinations and/or positive cultures from sputum, endotracheal tubes, or tracheostomies.

#### Follow-up data

Clinical follow-up was performed six weeks, six months and one year post trauma. The patients answered a standardized questionnaire concerning pain, local tenderness, breathlessness, and analgesia use. The dose of opioid medication used was recorded and the equivalent oral morphine dose calculated. Quality of Life (QoL) was assessed by using the five-graded EQ-5D-5L instrument [22] which poses questions concerning mobility, self-care, usual activities, pain/discomfort, and anxiety/depression. The results are converted to a single summary index using the Time Trade-Off (TTO) technique with a Swedish value set [23]. In addition, the patients grade their perceived health on a visual analogue scale (VAS) from 0–100. Reference values for EQ-TTO are 0.9 ± 0.2 and EQ-VAS 76.1 ± 18.7 from a Swedish population study [23]. Physical activity was assessed by using the Saltin-Grimby activity scale, where patients grade their activity; physically inactive (1), light physical activity (2), regular physical activity and training (3), hard physical training for competitive sports (4) [24, 25]. Physical function was assessed by using the Disability Rating Index (DRI) questionnaire where patients grade their answers to questions concerning different physical activities using VAS from 0 (i.e., no difficulty) to 100 (i.e., maximal difficulty) [26]. Type of work and return to work was recorded.

Lung function tests were performed in a standardized manner using an EasyOne**®** Spirometer (ndd Medical Technologies Inc., MA, US) and predicted Forced Vital Capacity (FVC), Forced Expiratory Volume in one second (FEV1) and Peak Expiratory Flow (PEF) were recorded [27]. Maximal Inspiratory Pressure (MIP) and Maximal Expiratory Pressure (MEP) were measured by MicroRPM™ (Respiratory Pressure Meter: Care Fusion, Sollentuna, Sweden). The range of motion in the thorax was assessed by measuring thoracic excursion, the difference in thoracic circumference between maximal inspiration and expiration at the level of the fourth rib and the level of the xiphoid process in an upright patient [28]. Movement of the thoracic spine was assessed by measuring flexion and extension by identifying the C7 spinous process and a point 30 cm below. The patient was asked to bend forwards and backwards with straight legs and the difference was recorded. [29] Lateral flexion was measured by having the patient stand upright against a wall and flex laterally [29]. Breathing movements at rest and during maximal breathing were measured by using a Respiratory Movement Measuring Instrument, RMMI**®** (ReMo Inc. Keldnaholt, Reykjavik, Iceland) [30, 31]. Range of motion in the shoulders was assessed by using a Goniometer and Boström index [32]. Patients underwent a CT examination with intravenous contrast medium of the thorax (often as part of a whole-body CT), upon arrival at the hospital before inclusion. The extent of injury to the chest wall and intra-thoracic injuries were assessed. A second CT examination of the thorax, without intravenous contrast medium, was performed six months post trauma in order to study CT-lung volume. Thoracic VCAR (Volume Computer Assisted Reading) Parenchyma Analysis imaging software (GE Healthcare, Waukesha, USA) was used to calculate CT-lung volume in litres from air-filled lung tissue in 0.6 mm thick CT images pre- and post-operatively, as previously described [33].

### Statistical analyses

Statistical analyses were done using SPSS v29.0.2 software (IBM**®** 2023). Results from normally distributed continuous variables are shown as mean with standard deviation (SD) and compared using independent 2-tailed T-test. Whereas, non-parametric data is shown as median with range or median with 5 th (P_5_) and 95 th (P_95_) percentiles, and compared using Mann–Whitney U Test. Categorical variables are shown as n and % and compared using the Pearson’s Chi-square Test and Fisher´s exact test. Bonferonni adjustment was used for subgroup analyses with Z test to correct for multiple tests. Significance was considered to be *p* < 0.05.

## Results

**Fig. 1:**
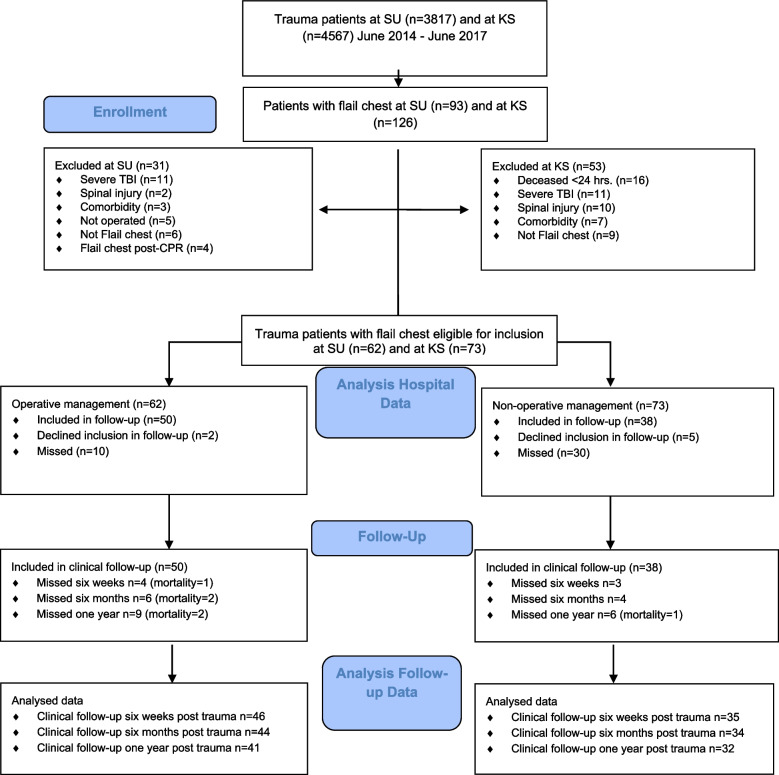
CONSORT flow diagram of patients included in the study. SU = Sahlgrenska University Hospital; KS = Karolinska University Hospital Solna; TBI = Traumatic Brain Injury

Two-hundred-nineteen trauma patients with unstable chest wall injuries were identified through hospital records and the Swedish National Trauma Registry (SweTrau) at Sahlgenska University Hospital and Karolinska University Hospital Solna during 2014–2017. Of these, 62 patients were eligible for inclusion in the Op group and 50 patients agreed to participate in a one-year follow-up, of which 96% (*n* = 48) attended. Seventy-three patients were eligible for inclusion in the Non-Op group, and 38 agreed to participate in a one-year follow-up, of which 92% (*n* = 35) attended (Fig. 1).

Patient demographics are shown in Table 1. There were proportionately more women in the Op group compared to the Non-Op group (27% vs 11%, *p* = 0.014), but no difference in age, smoking habits and preexisting lung disease between the groups. The distribution of MOI differed between the groups. There was no difference in ISS, however NISS and the distribution of AIS Head and Thorax differed between the groups with the Op group having higher NISS and more severe chest wall injuries and the Non-op group more head injuries.

**Table 1:** Demographics of patients managed operatively (Op group) and non-operatively (Non-Op group) for flail chest injuries

Variable	Included patients		
	**Op group** **(*n*=62)**	**Non-Op group** **(*n*=73)**	***p*-value**
**Sex** (n, %)			0.014
Male	45 (72.6)	65 (89.0)	
Female	17 (27.4)	8 (11.0)	
**Age**	59.1 ± 15.0	54.9 ± 16.1	0.124
**Smoker**			> 0.05
No	18 (36.0)	17 (44.7)	
Yes	17 (34.0)	6 (15.8)	
Previous	14 (28.0)	14 (36.8)	
Unknown	1 (2.0)	1 (2.6)	
**Lung disease**			0.979
No	42 (84.0)	32 (84.2)	
Yes	8 (16.0)	6 (15.8)	
**MOI** (n, %)			
MVC	13 (21.0)	3 (4.1)	< 0.05
Bicycle	7 (11.3)	11 (15.1)	> 0.05
Motorcycle	1 (1.6)	17 (23.3)	< 0.05
PVA	4 (6.5)	5 (6.8)	> 0.05
Fall same level	11 (17.7)	3 (4.1)	< 0.05
Fall from height	17 (27.4)	26 (35.6)	> 0.05
Crush injury	3 (4.8)	2 (2.7)	> 0.05
Assault	0 (0)	2 (2.7)	n/a
Miscellaneous	6 (9.7)	4 (5.5)	> 0.05
**ISS**			
Median (P_5_, P_95_)	21.0 (9.0, 41.9)	21.0 (10.0, 48.6)	0.979
**NISS**			
Median (P_5_, P_95_)	34.0 (22.0, 50.0)	29.0 (17.0, 57.0)	0.042
**Head AIS**			
0 (n, %)	49 (79.0)	41 (56.2)	< 0.05
1 (n, %)	5 (8.1)	9 (12.3)	> 0.05
2 (n, %)	2 (3.2)	16 (21.9)	< 0.05
3 (n, %)	6 (9.7)	7 (9.6)	> 0.05
**Thoracic AIS**			
3 (n, %)	23 (37.1)	44 (60.3)	< 0.05
4 (n, %)	29 (46.8)	26 (35.6)	> 0.05
5 (n, %)	10 (16.1)	3 (4.1)	< 0.05

Continuous variables are shown as mean with SD and compared with T-test or shown as median with P_5_ and P_95_ and compared with Mann–Whitney U test. Categorical variables are shown as number and per cent and compared with Pearson’s chi-square Test or Fisher’s Exact Τest, and subgroup analyses were performed with Z-test

*MOI* Mechanism of injury, *MVC* Motor vehicle collision, *PVA* Pedestrian vehicle accident, *ISS* Injury severity score, *NISS* New injury severity score, *AIS* Abbreviated injury scale, P_5_ = 5 th percentile, P_95_ = 95 th percentile, n/a = non applicable

### Hospital data outcome

Patients in the Op group underwent surgical fixation of the chest wall at a median of 4 days (range 2–14) post trauma and median 2 days (range 1–10) after the decision to operate was made. Twenty-nine per cent of patients required ventilatory support in both groups with a median LOS of 8.5 (range 1.0–42.0) and 8.0 (range 1.0–32.0) days in the Op group and Non-Op group, respectively. (Table 2). In the Op group 15 patients (24%) required MV pre-operatively for 3.0 (range 1.0–8.0) days and 17 patients (27%) required mechanical ventilation post-operatively for 4.0 (range 1.0–39.0) days. There was no statistical difference in the LOS on MV or in ICU between the two groups. Hospital LOS was longer in the Op group compared to the Non-Op group (median 14 vs. 11 days, *p* = 0.001). There was no significant difference in the proportion of patients receiving a tracheostomy between the groups. Pneumonia was diagnosed in 27 (37%) of patients in the Non-Op group compared to 11 (18%) of patients in the Op group (*p* = 0.013). In five cases, pneumonia was diagnosed pre-operatively in the Op group. Three patients in the Non-Op group developed empyema, one patient required a thoracotomy, and another was managed with video-assisted thoracoscopic surgery (VATS). None of the patients in the Op group developed empyema or had retained hemothorax that required additional management. However, we did not study the use of chest drains or retained hemothorax in detail. Two patients in the Op group required re-operation due to bleeding. There was no difference in 30-day and one-year mortality rate between the groups. Two patients in the Non-Op group were treated with Extracorporeal membrane oxygenation (ECMO) due to the development of severe respiratory insufficiency. Data on the use of TEDA was available for the patients included in the follow-up and there was no significant difference between the Op and Non-Op groups (71% vs 55%, *p* = 0.118).

**Table 2:** Hospital outcome measures of patients managed operatively (Op group) or non-operatively (Non-Op group) for flail chest injuries

**Variable**	**Op group** ( ***n*** ** = 62)**	**Non-Op group** ( ***n*** ** = 73)**	***p*** **-value**
Mechanical ventilator			
Need (n, %)	18 (29.0)	21 (29.2)	0.986
Days^a^ (median, P_5_, P_95_)	8.5 (1.0, 40.5)	8.0 (1.0, 31.3)	0.936
ICU care			
Need (n, %)	31 (50.0)	39 (53.4)	0.691
Days^a^ (median, P_5_, P_95_)	6.0 (1.0, 40.8)	5.0 (1.0, 31.2)	0.926
Hospital LOS			
Days (median, P_5_, P_95_)	14.0 (7.2, 44.9)	11 (2.7, 41.6)	0.001
Tracheostomy			
(n, %)	8 (12.9)	6 (8.2)	0.374
Pneumonia			
(n, %)	11 (17.7)^b^	27 (37.0)	0.013
Empyema			
(n, %)	0 (0)	3 (4.1)	
Re-operation			
(n, %)	2 (3.2)	n/a	
Mortality			
30-days (n, %)	2 (3.2)	2 (2.7)	0.868
One-year (n, %)	5 (8.1)	5 (6.8)	0.788

Continuous variables are shown as median with P_5_ and P_95_ and compared with Mann–Whitney U test. Categorical variables are shown as number and per cent and compared with Pearson’s chi-square Test or Fisher’s Exact Τest

*ICU* Intensive Care Unit, *LOS* Length of Stay, P_5_ = 5 th percentile, P_95_ = 95 th percentile, n/a = not applicable

^a^Analysis includes only patients with need of mechanical ventilation or intensive care, respectively

^b^Present pre-operatively (*n* = 5)

### Follow-up data outcome

Fifty patients in the Op group and 38 patients in the Non-Op group were included in a one-year follow up of which 48 and 35 patients attended, respectively (Fig. 1). There was a difference in age between the prospectively studied patients in the Op group and the Non-Op group, 59.3 ± 15.6 vs. 52.0 ± 13.6 (*p* = 0.023). Patients in the Op group had higher median NISS, 34 (20, 54) vs. 27 (14, 48) (*p* = 0.003). There was no significant difference in ISS, sex, smoking habits or pre-existing lung disease. The number of ribs fractured were 11.4 ± 4.3 in the Op group compared to 10.6 ± 3.8 in the Non-Op group (*p* = 0.357) and the number of fractured ribs was 20.3 ± 7.8 in the Op group compared to 19.0 ± 6.5 in the Non-Op group (*p* = 0.384). There were more patients with bilateral flail segments in the Op group (*n* = 5) compared to the Non-Op group (*n* = 2), but this difference was not significant. There was no statistical difference in the proportion of patients with pneumothorax (69 vs 83%), hemothorax (88 vs 77%), lung contusion (69%), lung laceration (17 vs 29%) or sternal fracture (25 vs 23%) between the Op group and the Non-Op group. There was a significant difference in AIS Thorax between the groups whereby nine patients had AIS 5 in the Op group and none in the Non-Op group. Median AIS Thorax was 4 (3–5) vs 3 (3–4) in the Op group and Non-Op group respectively (*p* = 0.009).

Patients in the Op group had less complaints concerning pain, local tenderness, problems with self-care and had a significantly lower intake of daily oral morphine, six weeks post trauma, compared to patients in the Non-Op group (Table 3). Patients in the Non-Op group experienced more pain when breathing, anxiety and depression six months post trauma. There was no significant difference in physical activity, physical function estimated by DRI, QoL Index and VAS estimated by EQ-5D5L and work activity between the groups at the follow-ups. Patients in the Op group returned to work 62 (18–262) days from trauma compared to 89 (5–342) days in the Non-Op group, this difference was not statistically significant (*p* = 0.208). After one year there were no significant differences between the two groups.

**Table 3:** Patient reported outcome measures six weeks, six months and one year post trauma, in patients managed operatively (Op group) and non-operatively (Non-Op group) for flail chest injuries

Symptoms	Six weeks		Six months		One year	
	**Op group ** ***n*** ** = 46**	**Non-Op group ** ***n*** ** = 35**	**Op group ** ***n*** ** = 44**	**Non-Op group ** ***n*** ** = 34**	**Op group ** ***n*** ** = 41**	**Non-Op group ** ***n*** ** = 32**
Pain at rest (n, %)	12 (26)	21 (60)*	5 (12)	7 (21)	2 (5)	2 (6)
Pain when breathing (n, %)	9 (20)	25 (71)*	5 (12)	11 (32)*	4 (10)	5 (16)
Local tenderness (n, %)	23 (50)	33 (94)*	20 (47)	16 (47)	15 (37)	12 (38)
Breathlessness (n, %)	17 (37)	18 (51)	10 (23)	12 (35)	6 (15)	9 (29)
Pain medication (n, %)	31 (67)	26 (74)	11 (26)	6 (18)	7 (17)	5 (16)
Morphine dose (median mg, P_5_, P_95_)	0 (0, 106)	10 (0, 130)*	0 (0, 37)	0 (0, 48)	0 (0, 0)	0 (0, 13)
Physical activity (median, P_5_, P_95_)	4.0 (1.0, 4.0)	3.0 (1.0, 4.4)	4.0 (2.0, 6.0)	4.0 (1.8, 6.0)	4.0 (2.0, 6.0)	4.0 (2.0, 6.0)
DRI (median, P_5_, P_95_)	44.4 (12.2, 81.4)	46.8 (17.8, 83.2)	20.5 (0, 60.2)	11.1 (0, 69.8)	19.7 (0, 53.0)	18.9 (0, 63.5)
EQ-5D-5L problems:						
Mobility (n, %)	21 (46)	21 (60)	13 (30)	9 (27)	7 (17)	9 (28)
Self-Care (n, %)	18 (39)	22 (63)*	8 (19)	5 (15)	8 (20)	7 (22)
Usual Activities (n, %)	39 (85)	32 (91)	29 (67)	18 (53)	16 (39)	17 (53)
Pain/Discomfort (n, %)	42 (91)	34 (97)	36 (84)	32 (94)	30 (73)	24 (75)
Anxiety/Depression (n, %)	23 (50)	20 (57)	12 (28)	17 (50.0)*	16 (39)	15 (47)
Index (mean ± SD)	0.66 ± 0.19	0.66 ± 0.12	0.76 ± 0.12	0.76 ± 0.13	0.78 ± 0.14	0.77 ± 0.15
Perceived Health (mean ± SD)	63.4 ± 19.3	61.6 ± 17.0	71.4 ± 17.5	76.8 ± 18.5	74.8 ± 14.6	77.1 ± 18.1
Working^a^	9 (32)	7 (26)	24 (86)	24 (92)	25 (89)	25 (100)
Non-physical (n, %)	4 (80)	5 (56)	5 (100)	8 (89)	5 (100)	8 (100)
Physical (n, %)	3 (23)	2 (22)	10 (77)	8 (89)	11 (85)	9 (100)
Heavy physical (n, %)	2 (20)	0 (0)	9 (90)	7 (100)	9 (90)	8 (100)

Continuous variables are shown as mean with SD and compared with T-test or shown as median with P_5_ and P_95_ and compared with Mann–Whitney U test. Categorical variables are shown as number and per cent and compared with Pearson’s chi-square Test or Fisher’s Exact Τest

*DRI* Disability Rating Index, *VAS* Visual Analogue Scale, P_5_ = 5 th percentile, P_95_ = 95 th percentile

^*^ *p* < 0.05

^a^Includes only patients that worked pre-injury within each group and attended each follow-up

There was no statistical difference in predicted FVC between the Op group and Non-Op group at six weeks, six months and one year follow-up (Table 4). Predicted FEV1 and MIP and MEP were consistently better in the Non-Op group. Thirty-four patients in the Op group had undergone thoracotomy with 11 having minor lung resection and/or suture repair, whilst 14 patients were managed with a muscle-sparing approach and without thoracotomy. Patients operated with thoracotomy had significantly worse lung function at follow-up, compared to patients managed non-operatively or operatively without thoracotomy. Patients operated with a muscle-sparing technique without thoracotomy had the best lung function, however this difference was not significant when comparing to the Non-Op group. Estimated CT-lung volume six months post trauma was 6.6 L in patients operated without thoracotomy compared to 5.3 L in patients operated with thoracotomy (*p* < 0.05). Comparison of CT-lung volume on initial CT with CT after six months, showed significant improvement, regardless of whether the patient had been intubated at the time of initial CT or if an operation had been performed, with or without thoracotomy. However, the increase in CT-lung volume in patients in the Op group without thoracotomy was significantly better than in patients in the Non-Op group (3.3 vs. 2.7 L, *p* = 0.028) or in patients in the Op group with thoracotomy (3.3 vs. 2.9 L, *p* = 0.041). No difference in improvement was seen between the two latter groups. There was no significant difference in pneumothorax volume at initial CT that would account for the difference.

**Table 4:** Thoracic injuries, estimated CT-lung volume, and lung function 6 weeks, 6 months and one year post-trauma in patients managed operatively (Op group) with or without a thoracotomy, and non-operatively (Non-Op group) for flail chest injuries

Follow-up	Variable	Op group			Non-Op group
		All (*n* = 48)	+ Thoracotomy (*n* = 34)	-Thoracotomy (*n* = 14)	(*n* = 35)
Initial CT	CT Lung volume (l)	2.72 (1.30–5.31)	2.56 (1.43–4.69)	3.31 (1.30–5.31)	2.92 (1.69–6.97)
	Pneumothorax (n, %)	33 (69)	25 (74)	8 (57)	29 (83)
	Pneumothorax volume (l)	0.22 (0.01–1.02)	0.13 (0.01–0.62)	0.76 (0.50–1.02)	0.11 (0.02–0.62)
	Bilat Flail chest (n, %)	5 (10)	4 (12)	1 (7)	2 (6)
	Hemothorax (n, %)	42 (88)	30 (88)	12 (86)	27 (77)
	Lung contusion (n, %)	33 (69)	24 (71)	9 (64)	24 (69)
	Lung laceration (n, %)	8 (17)	5 (15)	3 (21)	10 (29)
6 weeks	Predicted FVC (%)	84 (47–125)	78 (47–99)^a,b^	92 (54–125)	89 (47–111)
	Predicted FEV1 (%)	76 (42–140)^a^	67 (42–95)^a,b^	92 (59–140)	84 (39–113)
	Predicted PEF (%)	88 (42–142)	82 (47–132)^b^	103 (42–142)	101 (27–134)
	MIP (cm H_2_0)	70 ± 29^a^	64 ± 28^a,b^	84 ± 28	87 ± 27
	MEP (cm H_2_0)	98 ± 35^a^	94 ± 29^a^	106 ± 47	130 ± 37
6 months	CT Lung volume (l)	5.61 (2.11–8.77)	5.30 (2.11–7.40)^b^	6.60 (3.87–8.77)	5.80 (1.82–8.04)
	Predicted FVC (%)	88 (41–138)	83 (41–112)^b^	96 (70–138)	94 (47–118)
	Predicted FEV1 (%)	81 (39–120)^a^	75 (39–103)^a,b^	96 (69–120)	94 (45–131)
	Predicted PEF (%)	89 (41–148)^a^	81 (41–148)^a,b^	97 (41–131)	100 (33–150)
	MIP (cm H_2_0)	78 ± 36^a^	74 ± 37^a^	89 ± 35	104 ± 28
	MEP (cm H_2_0)	107 ± 39^a^	104 ± 39^a^	114 ± 42^a^	155 ± 49
1 year	Predicted FVC (%)	90 (49–138)	82 (49–115)	97 (76–138)	96 (48–118)
	Predicted FEV1 (%)	82 (39–126)^a^	75 (39–108)^a,b^	96 (72–126)	96 (41–123)
	Predicted PEF (%)	98 (43–134)	93 (43–134)	107 (70–129)	108 (25–155)
	MIP (cm H_2_0)	82 ± 31^a^	78 ± 28^a^	95 ± 37	106 ± 29
	MEP (cm H_2_0)	110 ± 39^a^	108 ± 38^a^	116 ± 44^a^	158 ± 43

Continuous variables are shown as mean with SD and compared with T-test or shown as median with range and compared with Mann–Whitney U test

*CT* Computed Tomography, *FVC* Forced Vital Capacity, *FEV1* Forced Expiratory Volume in 1 s, *PEF* Peak Expiratory Flow, *MIP* Maximal Inspiratory Pressure, *MEP* Maximal Expiratory Pressure

^**a**^Difference between Op group and Non-Op group (*p* < 0.05)

^**b**^Difference within Op group with and without thoracotomy (*p* < 0.05)

Range of motion in the thorax and shoulders, and breathing movements were compared in patients managed operatively and non-operatively (Table 5). Patients with bilateral flail segments were excluded from analyses concerning comparison of shoulder function and breathing movements, between injured and non-injured sides. Upper thoracic excursion was consistently better in the Non-Op group, 5.1 cm compared to 3.3 cm (*p* < 0.05) one year post trauma. Thoracic flexion was better in the Op-group, but only significantly at six weeks follow-up. Lateral flexion at six months and one year was better in the Non-Op-group. There were no significant differences in shoulder function or breathing movements between the groups.

**Table 5:** Comparison of range of motion in the thorax and shoulders, and breathing movements at rest and during maximal breathing, six weeks, six months and one-year post-trauma, in patients managed operatively (Op-group) and non-operatively (NonOp-group) for flail chest injuries

Symptoms	Six weeks		Six months		One year	
	**Op-group** ***n*** ** = 46**	**NonOp-group** ***n*** ** = 35**	**Op-group** ***n*** ** = 44**	**NonOp -group** ***n*** ** = 34**	**Op-group** ***n*** ** = 41**	**NonOp -group** ***n*** ** = 32**
Upper thoracic excursion (cm)	3.3 ± 1.5	4.4 ± 1.4*	3.5 ± 1.6	4.9 ± 2.0*	3.3 ± 1.8	5.1 ± 1.9*
Lower thoracic excursion (cm)	4.0 ± 1.9	4.6 ± 1.4	4.4 ± 2.5	4.9 ± 1.7	4.2 ± 2.5	4.6 ± 1.7
Thoracic flexion (cm)	2.2 ± 0.8*	1.3 ± 0.7	1.9 ± 0.9	1.7 ± 1.1	2.0 ± 1.0	1.6 ± 1.0
Thoracic extension (cm)	1.1 ± 0.8	1.4 ± 0.6	1.2 ± 0.7	1.5 ± 1.0	1.1 ± 0.6	1.4 ± 0.8
Lat. flex. injured (cm)^a^	13.4 ± 5.3	13.7 ± 5.7	13.3 ± 4.8	16.0 ± 4.2*	13.1 ± 6.3	16.7 ± 4.5*
Lat. flex. non-injured (cm)^a^	12.8 ± 4.5	14.2 ± 4.8	13.5 ± 4.6	16.8 ± 4.0*	13.8 ± 5.3	17.1 ± 4.5*
Flex. shoulder injured (0–180^°^)^a^	134.5 ± 41.2	130.8 ± 45.1	151.5 ± 30.8	159.7 ± 17.4	151.4 ± 32.1	159.5 ± 12.1
Flex. shoulder non-injured (0–180^°^)^a^	156.3 ± 27.1	161.4 ± 12.9	161.0 ± 25.4	164.8 ± 13.5	165.3 ± 20.4	160.3 ± 17.7
Abd. shoulder injured (0–180^°^)^a^	132.1 ± 45.8	129.4 ± 43.9	145.8 ± 40.5	159.1 ± 24.5	146.7 ± 33.4	160.2 ± 17.1
Abd. shoulder non-injured (0–180^°^)^a^	152.7 ± 32.8	156.5 ± 20.8	153.8 ± 35.2	165.2 ± 16.3	158.6 ± 29.5	158.8 ± 23.5
Boström score	50.0 ± 9.8	47.6 ± 8.9	53.2 ± 7.7	54.2 ± 5.7	54.6 ± 6.7	54.4 ± 7.9
Breathing Movements at Rest^a^						
Upper Thorax (Injured/Non-Injured%)	83.8 ± 19.8	90.8 ± 18.7	89.8 ± 34.1	98.9 ± 30.1	91.2 ± 25.3	95.5 ± 20.5
Lower Thorax (Injured/Non-Injured%)	90.6 ± 23.1	97.1 ± 31.3	88.3 ± 34.5	96.7 ± 29.7	94.6 ± 33.0	110.3 ± 40.8
Abdomen (Injured/Non-Injured%)	89.6 ± 23.8	97.4 ± 13.9	95.1 ± 17.8	98.9 ± 12.3	99.0 ± 36.4	100.1 ± 15.0
Breathing Movements at Maximal^b^						
Upper Thorax (Injured/Non-Injured%)	85.5 ± 22.0	87.0 ± 13.0	91.7 ± 19.2	99.8 ± 21.6	87.6 ± 21.4	96.0 ± 16.3
Lower Thorax (Injured/Non-Injured%)	92.2 ± 49.9	90.9 ± 20.7	97.0 ± 43.3	98.8 ± 18.9	92.2 ± 37.4	98.4 ± 17.1
Abdomen (Injured/Non-Injured%)	92.1 ± 20.3	98.8 ± 17.2	97.6 ± 23.0	103.6 ± 16.8	95.5 ± 20.9	100.6 ± 15.5

Continuous variables are shown as mean with SD and compared with T-test

*Lat* Lateral, *Flex* Flexion, *Abd* Abduction

^a^Measurements exclude patients with bilateral flail chest

^*^ *p* < 0.05

## Discussion

In this bi-institutional cohort study we compared operative and non-operative management of flail chest injuries between the two largest trauma centers in Sweden. We found that 2.6% of all registered trauma patients had flail segments and/or clinical flail chest. There was no difference in the need for MV or LOS on ventilator and in ICU between patients in the Op group and the Non-Op group. Twenty-nine per cent of the patients required MV in our study. This is in contrast to a previous study from Sweden showing the need for MV in 47% of patients [19]. However, the latter study also included patients with severe head injuries. Previous studies have shown a decreased LOS on MV [4–6] and in ICU [4–7] in patients managed operatively for flail chest injuries. Our cohort consisted of patients with both clinical flail and radiological flail and not all patients needed ventilation at time of inclusion which may influence the difference in results compared to previous studies and our primary endpoint.

The Op group had longer LOS in hospital than the Non-Op group. There was no significant difference in the number of rib fractures between the groups but there was a higher frequency of bilateral flail injuries and a significantly higher NISS in the Op group and these patients were also significantly older. Previous studies have shown that patients operated 24–72 h post trauma have fewer days of MV, lower ICU and hospital LOS, and.

decreased incidence of pneumonia and tracheostomy [1, 5, 34]. In our study, median day of operation was 4 days post-trauma with a range of 2–14 days. The reason for delay in surgery was late referral to the regional trauma center, possibly delay in decision to operate and an additional, median 2 days after decision was made until operation was performed. The delay in operation in this study could have influenced our primary endpoint and our secondary outcome measures. However, it is not always possible to operate a patient for unstable chest wall injuries within 48 h post trauma, unless the patient has an isolated thoracic injury. Most poly-trauma patients have other injuries that may take priority and the physiology needs to be in balance, which may take 24–48 h in severely injured patients, before chest wall stabilization can ensue [35].

We found pneumonia to be more common in the non-operatively managed patients, which is consistent with other studies [1, 4–6, 14, 34]. Two patients in the Non-Op group and none in the Op group developed empyema indicating some benefit with surgery and possibly thoracotomy, in performing a pleural toilet. However, we did not include the study of chest drains or retained hemothorax in our study protocol, but none of the patients in the Op group required additional drain or treatment. It is possible that an early operation, preventing the development of pneumonia, would have influenced this outcome as half the cases of pneumonia in the Op group were diagnosed pre-operatively. Two patients managed non-operatively were treated with ECMO for respiratory insufficiency. None of the patients managed operatively, were treated with ECMO. However, these results are difficult to compare as the protocols for using ECMO in trauma patients differ between the two hospitals. There was no difference in the incidence of tracheostomy which is related to the length of stay on mechanical ventilator.

The operative technique with 70% of patients undergoing thoracotomy in the Op group may also influence both short- and long-term outcome. Surgery without thoracotomy was associated with the best lung function and CT lung volume at follow-up, although the difference with non-operatively managed patients was not significant, surgery with thoracotomy was associated with the worst results. However, it is possible that this result not only reflects the morbidity caused by the procedure but also, that these patients had more severe intra-thoracic injuries, as a decision to perform a thoracotomy was made. Maximal Expiratory Pressure was significantly higher in non-operatively managed patients compared to operatively managed patients, regardless of whether a thoracotomy had been performed. This may suggest a negative effect of chest wall surgery on the expiratory muscles but may also be related to the differences in age between the groups.

Computed tomography estimates of lung volume increased significantly when comparing initial CT to CT after six months, in all groups of patients. As seen previously, intubation at initial CT did not change this outcome [33]. The improvement in CT lung volume, which has a high correlation to Total Lung Capacity (TLC) [33], was significantly higher in patients managed operatively without a thoracotomy, indicating a positive effect of chest wall stabilization with a muscle-sparing minimally invasive approach on lung volume.

We found that operatively managed patients had less pain and required a significantly lower dose of oral morphine the first months’ post trauma, compared to non-operatively managed patients. It is possible that surgery has the largest impact on pain the first weeks post trauma and that we would have seen differences consistently between the groups if we had included earlier time points in the study protocol. Previous studies have also shown differences in pain experience in the first weeks to months post trauma [4, 6, 14, 15], whilst others have found no difference between operatively and non-operatively managed patients [5, 7]. Surgical approach and type of implant might influence this experience. Despite operatively managed patients in our study being significantly older and with a higher thoracic injury burden, no difference was seen between the groups concerning physical activity, physical function and QoL. None of the non-operatively managed patients had returned to heavy physical work within the first six weeks post trauma. After one year, there was no significant difference between operatively and non-operatively managed patients concerning subjective symptoms, function, disability, QoL or work activity. The EQ-VAS was comparable to a normal population after one year [23]. Extra-thoracic injuries may well influence these results as well as long-term outcome and although we excluded patients with severe TBI and spinal injury we did not study other injuries separately apart from the calculation of ISS and NISS.

Patients were assessed concerning range of motion in the shoulders, thoracic spine and chest wall. The Non-Op group had consistently better movement in upper thoracic excursion and also lateral flexion both on the injured and non-injured side suggesting more rigidity in the Op group. This could also be exacerbated by the high frequency of thoracotomies but could also be the results of multiple plate fixation. We did not analyze data from patients undergoing minimally invasive surgery separately from those undergoing a more extensive and invasive procedure. There was no long-term difference in outcome concerning range of motion in the thoracic spine and shoulders, shoulder function or breathing movements between the groups.

There are several limitations to this study. The inclusion to the follow-up study was terminated before statistical power was reached concerning the primary outcome variable as one of the centers began operating selected patients and there was concern for selection bias. Therefore, data on hospital outcome was added retrospectively from additional eligible patients treated during the inclusion period. Only 50% of eligible patients in the Non-Op group participated in the prospective follow-up. There was a drop-out in both groups of 4–8%. The drop-out was bigger in the Non-op group which could influence the results as the most healthy patients may refrain from attending clinic if they feel well and the most injured may not be able to attend the clinic. The patients were not completely comparable, as Thoracic AIS and NISS were higher in the Op group and Head AIS higher in the Non-Op group. Patients included in the prospective cohort were significantly older in the Op group. Some patients were transferred from other hospitals, as both hospitals serve as regional trauma centers. However, despite operation ≥ 48 h, higher Thoracic AIS and NISS, there was a reduced incidence of pneumonia and less pain in operated patients. The surgical technique changed during the study from more invasive surgery with thoracotomy to more muscle-sparing surgery. This could influence the outcome as the treatment was not standardized and a delay in operation may decrease the benefits from surgery. Another limitation is that there were no standardized protocols for the care of flail chest patients at the hospitals during the study period which might influence hospital outcome variables. Several years have passed since the collection of data and although development has taken place that may influence the hospital outcome variables, we believe the data from the long-term follow-up is valuable. The study includes data and multiple comparisons on two relatively small cohorts increasing the risk of statistical uncertainty and adjustment was only used for subgroup analysis.

In this study of ventilated and non-ventilated patients with clinical and radiological flail chest injuries we have compared short- and long-term outcome of operative and non-operative management. We have performed a detailed follow-up including patient-reported outcome measures, QoL, lung function, lung volume, respiratory muscle strength and movement of the shoulders, thoracic spine and chest wall. This is the first study, to our knowledge looking at all these variables and comparing CT-lung volumes between patients managed operatively, with or without thoracotomy, and non-operatively for flail chest injuries. We suggest further research concerning improvement of operative technique with more minimally invasive surgery for chest wall stabilization. Too few patients in our study were managed with muscle sparing minimally invasive technique to perform a subgroup analysis but it is possible that operative technique matters and may influence outcome [36].

## Conclusions

We found no difference in the need for or LOS on mechanical ventilator between operatively and non-operatively managed patients with clinical and radiological flail chest injuries. Despite no reduction in need of ventilation there may still be some benefit in operating non-ventilated patients with flail segments as they have a decreased incidence of pneumonia, less pain and subjective symptoms compared to non-operatively managed patients in the first months post trauma. No considerable differences in physical activity, physical function and QoL are seen between patients managed operatively vs. non-operatively after one year. Minimally invasive surgery without thoracotomy provides the best residual lung function and CT-lung volume and the influence of operative technique on outcome needs to be studied further.

## Data Availability

The data supporting the conclusions of this study are included within the article. The datasets generated and analysed during the current study are not publicly available for the protection of study subjects. Additional information can be provided from the corresponding author on request.

## Abbreviations

AIS: Abbreviated Injury Scale
CT: Computed Tomography
DRI: Disability Rating Index
FEV1: Forced Expiratory Volume in one second
FVC: Forced Vital Capacity
ICU: Intensive care unit
ISS: Injury Severity Score
LOS: Length of Stay
MEP: Maximal Expiratory Pressure
MIP: Maximal Inspiratory Pressure
MOI: Mechanism of injury
MV: Mechanical Ventilation
NISS: New Injury Severity Score
NSAID: Non-Steroidal Anti-Inflammatory Drugs
PEF: Peak Expiratory Flow
QoL: Quality of Life
RCT: Randomized Controlled Trial
RMMI: Respiratory Movement Measuring Instrument
SSRF: Surgical stabilization of rib fractures
TBI: Traumatic brain injury
TEDA: Thoracic Epidural Analgesia
TTO: Time Trade-Off
VAS: Visual analogue scale
VATS: Video-assisted thoracoscopic surgery
